# Diagnosis and treatment of acute Essex-Lopresti injury: focus on terminology and review of literature

**DOI:** 10.1186/s12891-018-2232-2

**Published:** 2018-08-29

**Authors:** Maurizio Fontana, Marco Cavallo, Graziano Bettelli, Roberto Rotini

**Affiliations:** 1 Orthopaedic Department, Infermi Hospital, Viale Stradone 9, 48018 Faenza, Italy; 2 Shoulder and Elbow Unit, IRCCS - Istituto Ortopedico Rizzoli, Via G.C. Pupilli 1, Bologna, Italy

**Keywords:** Acute Essex-Lopresti injury, Elbow, Wrist, Forearm instability

## Abstract

**Background:**

Acute Essex-Lopresti injury is a rare and disabling condition of longitudinal instability of the forearm. When early diagnosed, patients report better outcomes with higher functional recovery. Aim of this study is to focus on the different lesion patterns causing forearm instability, reviewing literature and the cases treated by the Authors and to propose a new terminology for their identification.

**Methods:**

Five patients affected by acute Essex-Lopresti injury have been enrolled for this study. ELI was caused in two patients by bike fall, two cases by road traffic accident and one patient by fall while walking. A literature search was performed using Ovid Medline, Ovid Embase, Scopus and Cochrane Library and the Medical Subject Headings vocabulary. The search was limited to English language literature. 42 articles were evaluated, and finally four papers were considered for the review.

**Results:**

All patients were operated in acute setting with radial head replacement and different combinations of interosseous membrane reconstruction and distal radio-ulnar joint stabilization. Patients were followed for a mean of 15 months: a consistent improvement of clinical results were observed, reporting a mean MEPS of 92 and a mean MMWS of 90.8. One case complained persistent wrist pain associated to DRUJ discrepancy of 3 mm and underwent ulnar shortening osteotomy nine months after surgery, with good results.

**Discussion:**

The clinical studies present in literature reported similar results, highlighting as patients properly diagnosed and treated in acute setting report better results than patients operated after four weeks. In this study, the definitions of “Acute Engaged” and “Undetected at Imminent Evolution” Essex-Lopresti injury are proposed, in order to underline the necessity to carefully investigate the anatomical and radiological features in order to perform an early and proper surgical treatment.

**Conclusions:**

Following the observations, the definitions of “Acute Engaged” and “Undetected at Imminent Evolution” injuries are proposed to distinguish between evident cases and more insidious settings, with necessity of carefully investigate the anatomical and radiological features in order to address patients to an early and proper surgical treatment.

## Background

The forearm can be considered as a single articulating unit where the close interdependence of multiple anatomical structures allows forearm rotation, elbow and wrist motion [1, 2]. All of these functions, especially pronation and supination, explain the complex integrated relationship between the bones and soft tissue along the entire length of this anatomical district. The forearm constraints are formed by the Proximal Radio-Ulnar Joint (PRUJ) mainly represented by the Radial Head (RH), the Interosseous Membrane (IOM) particularly in its central and stronger part named Central Band or Interosseous ligament (IOL) [3–5] and Distal Radio-Ulnar joint (DRUJ) represented by the Triangular Fibrocartilage Complex (TFCC) and, when present, by the Distal Oblique Band (DOB). All these anatomic and functional structures can be grouped under the name of the Forearm Unit [6]. In 1951 Peter Essex-Lopresti described the proximal migration of the radius following the surgical excision of comminuted RH fracture [7]. This longitudinal migration of the radius can generate when a traumatic axial load is transmitted from the wrist to the elbow, causing the combination of DRUJ disruption, rupture of the IOM and RH fracture. After Essex-Lopresti detailed description, this injury pattern gained the eponym of Essex-Lopresti Injury (ELI) [8]. Like other traumatic patterns, this lesion can be classified in the group of unstable fractures of the forearm, characterized by fracture of one or both forearm bones associated with lesion of some forearm main constraints (TFCC, IOM and RH). The lack of at least two constraints can lead to two different acute patterns: a typical ELI or a hidden form, difficult to be detected but still considerably harming the patient’s quality of life [9]. The development of these conditions depend from the IOM/TFCC reaction to the energy-related trauma. These lesions are often misdiagnosed in emergency room and not properly treated, leading to a Chronic ELI, a disabling condition extremely difficult to treat with positive outcomes [4, 9–15].

Aim of this work is to focus on the different lesion patterns causing forearm instability, reviewing literature and the cases treated by the Authors and to propose a new terminology for their identification.

## Methods

A literature search was performed using Ovid Medline, Ovid Embase, Scopus and Cochrane Library and the Medical Subject Headings vocabulary. The following terms were combined with ‘AND’ and ‘OR’: ‘essex’; ‘lopresti’; ‘acute’. A total of 42 articles were The search was limited to English language literature. Papers published before 2018 and clearly reporting clinical results and ELI treatment in acute setting were considered. A total of 4 articles were finally considered for the review.

For this study all the thirty-two patients affected by ELI who came to the Authors' attention between 2010 and 2016 have been retrospectively reviewed. Adams et al. considered the acute setting within four weeks from trauma, [8] and following this indication five patients have been selected for this study. All patients were males, with mean age of 40 years. The primary injury causing ELI was by bike fall in two patients, road traffic accident in two cases and fall while walking in one case.

**Fig. 1:**
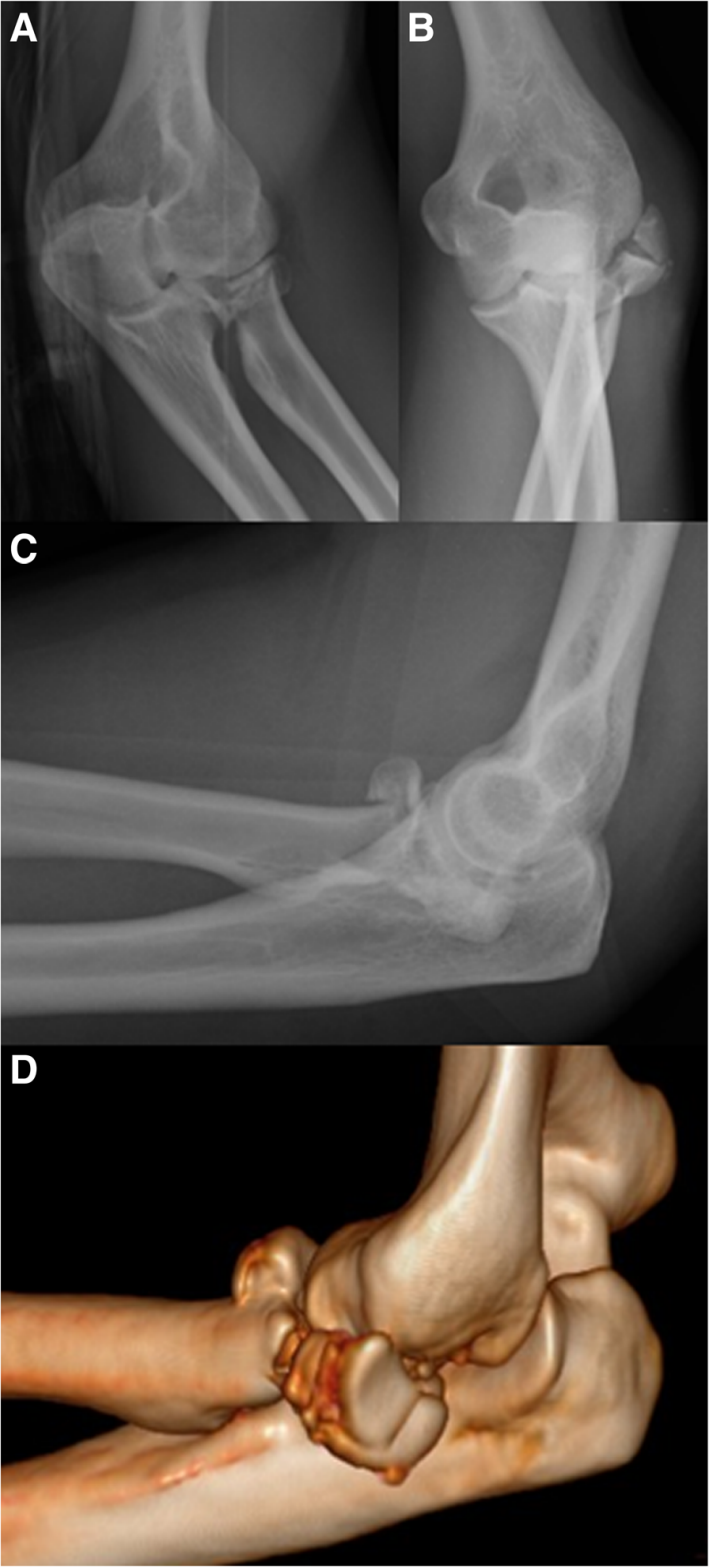
Clinical case 4, Acute Engaged ELI, pre-operative: elbow. Pre-operative left elbow X-rays (**a**, **b**, **c**) and 3D reconstruction CT scan (**d**) images showing a Mason 3 radial head fracture

**Fig. 2:**
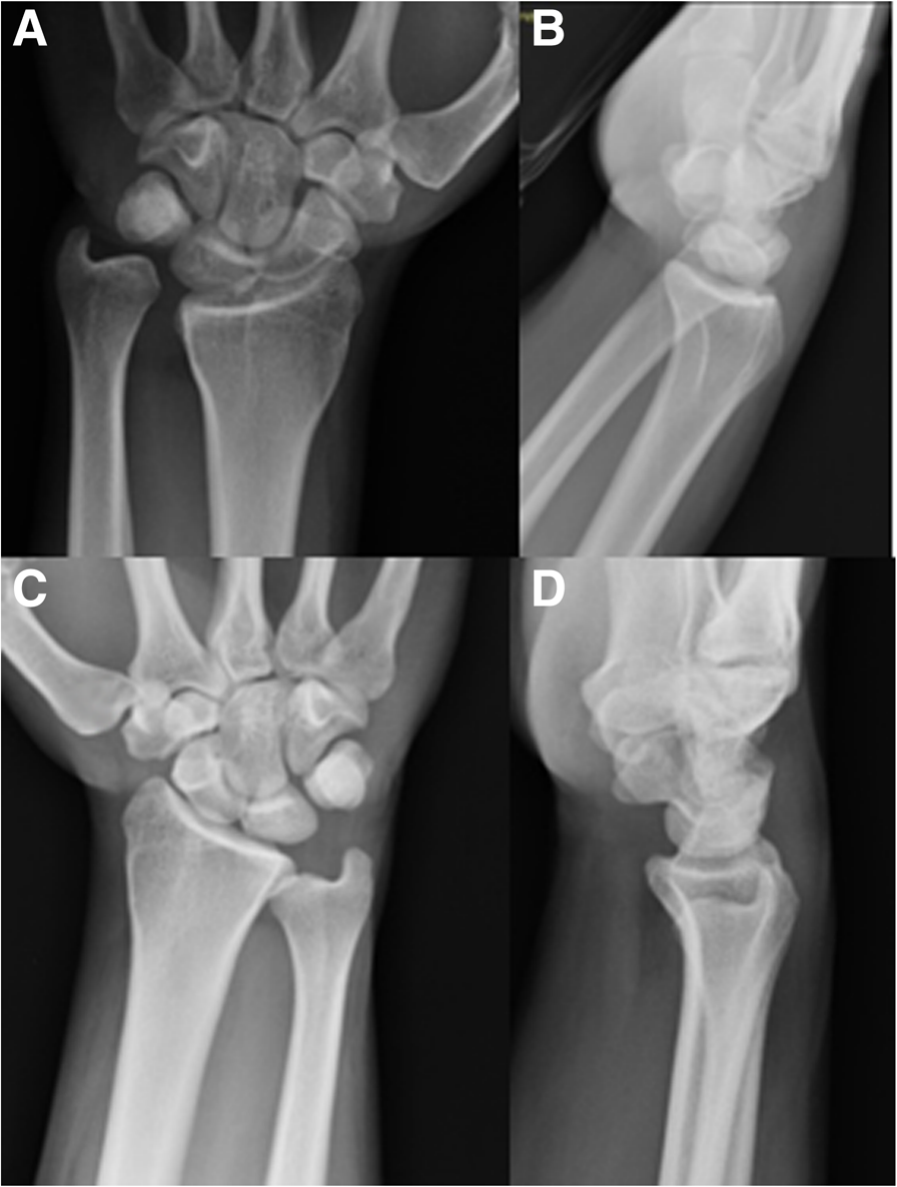
Clinical case 4, pre-operative: wrist. Pre-operative X-rays of the same patient. The left wrist (**a**, **b**) highlighted a DRUJ lesion, more evident if compared to the right unaffected wrist images (**c**, **d**)

**Fig. 3:**
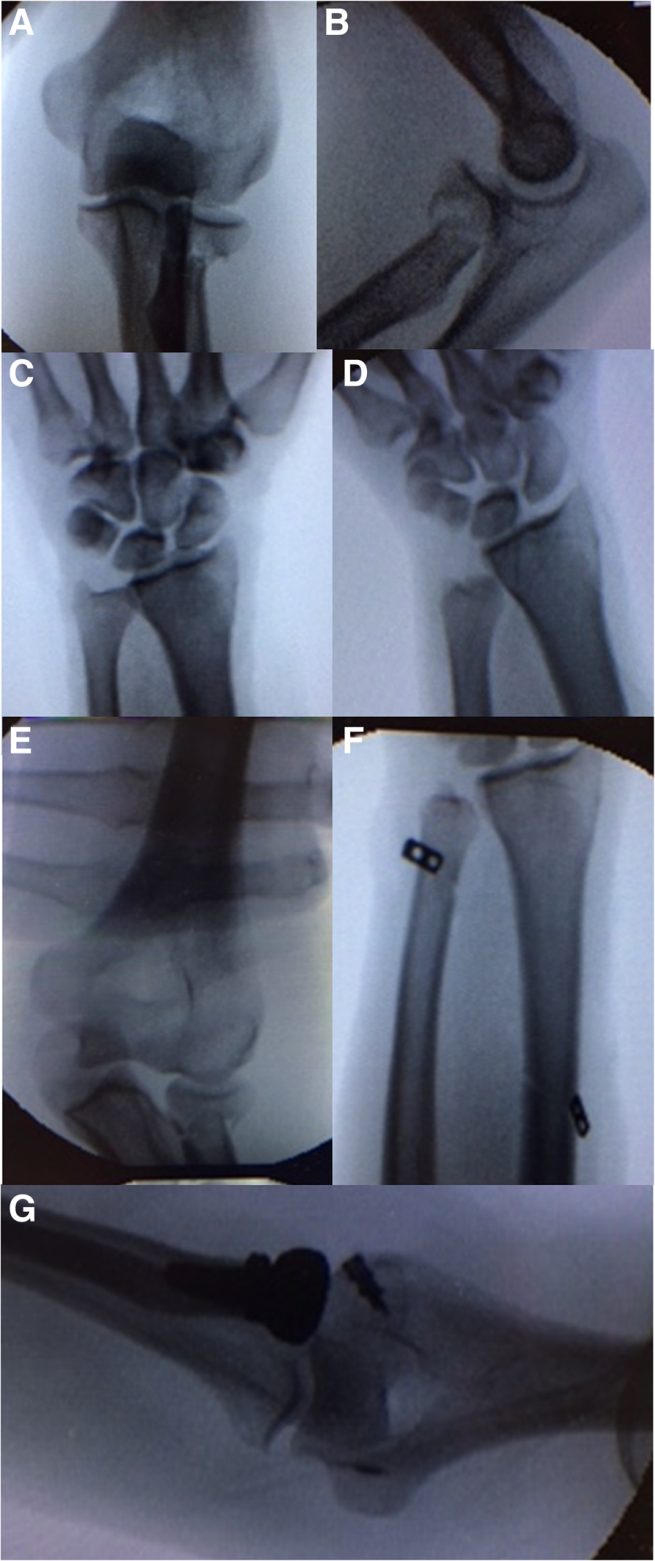
Clinical case 2: Acute Undetected at Imminent Evolution ELI pattern. Pre-operative X-ray of case n. 2: it is evident the radial head fracture without evident signs of high energy trauma (**a**, **b**). The DRUJ seemed aligned ad regular X-Ray (**c**). Performing the stress test under C-arm view the forearm longitudinal instability was detected (**d**, **e**). The treatment consisted in radial head prosthesis positioning (**g**), IOM plasty and collateral ligaments reconstruction (**f**)

Three cases presented an important proximal longitudinal dislocation of the radius, with the proximal radius engaging into the capitellum (Figs. 1, 2). In one case the RH fracture showed the involvement of radial neck (Mason grade 3) without longitudinal radial proximal dislocation, but in presence of gross instability of elbow and forearm (Fig. 3). In all the cases the lesion was caused by high energy upper limb impact trauma (bike fall, road traffic accident, and so on [16]. Patients’ demographics and lesion characteristics are reported in Table 2.

**Fig. 4:**
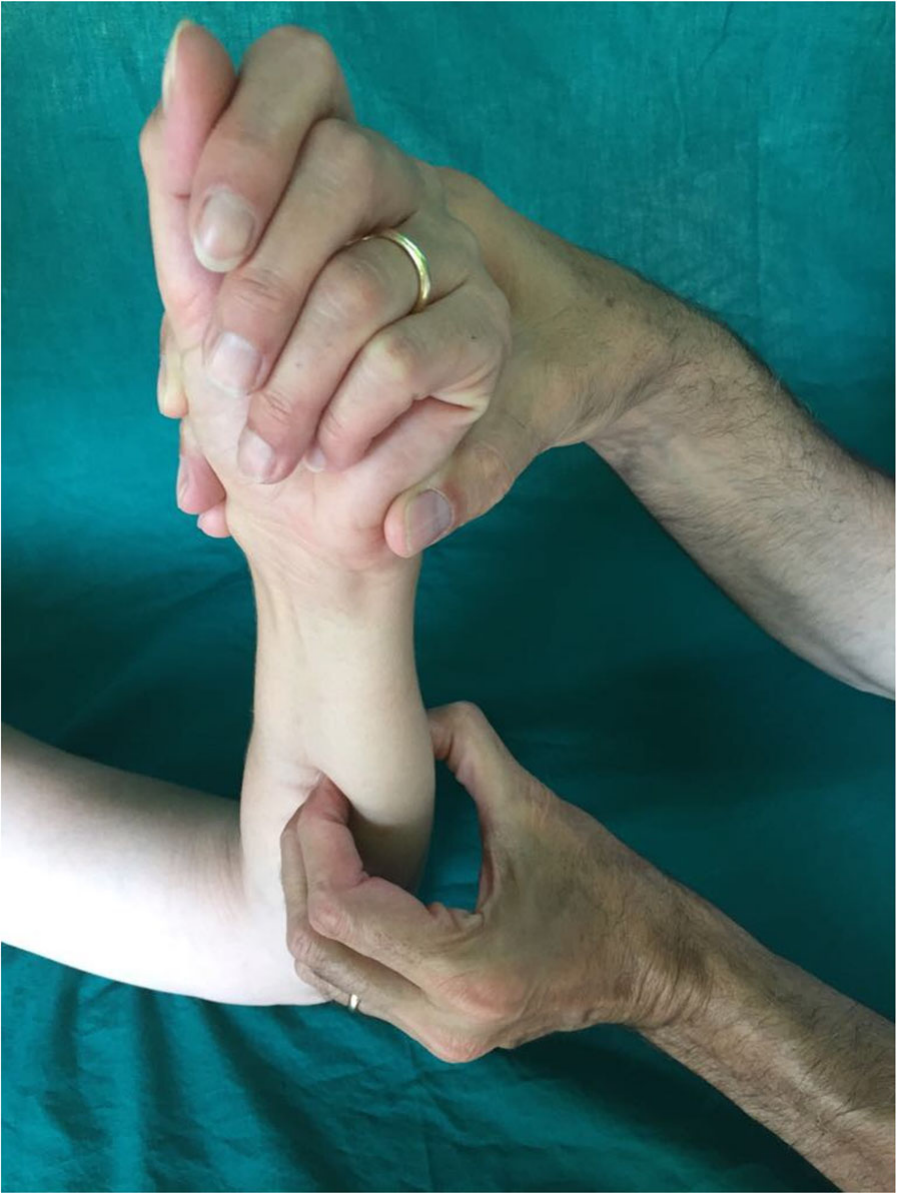
C-Fingers comparative test. Clinical image of the C-Fingers comparative test: the arm lies on a table, with elbow flexed at 90° and forearm vertical to the floor plane. With the thumb opposite to other fingers (forming the shape of a “C” letter) the surgeon squeezes the forearm space and pushes alternatively in dorsal and palmar direction to feel the muscular-IOM resistance in pronation and supination; the test must be comparative and is generally hindered by muscular hypertrophy and edema

The preliminary evaluation consisted in a clinical complete examination. In particular the investigation of the traumatic mechanism reported by the patient arose the suspect of high energy axial load on the forearm, with possibility of unstable fracture. The clinical examination was performed starting from the elbow stability evaluation (associated lesions of LUCL or MCL), followed by a check of the radial head (tenderness, pronation supination, Xilo Test). The IOM was checked with the “C-Fingers comparative test” [17] (Fig. 4): with this test it is possible to check the tenderness at the level of CB and DOB. In acute cases a vivid painful reaction is indicative of an IOM laceration. In chronic patients a reduced resistance of one or more segments compared to the counterlateral forearm is suspect for partial or complete IOM tear. The DRUJ was evaluated by the mean of the Tilt test: at the wrist the physician tests the DRUJ with dorsal and volar comparative translation of the ulna in neutral, supination and pronation. Then the potential longitudinal forearm instability was investigated with a comparative wrist X ray, with the detection of a distal radius proximal migration comparing to the counterlateral wrist. An elbow CT scan was performed in all cases to better assess the pathoanathomy of the RH fracture.

Surgery has been performed at a mean of 13 days after trauma. Before the surgical procedure the ELI was confirmed under anesthesia, performing some specific tests to better assess the elbow stability: the ultrasonographic evaluation of the so called “Muscular Hernia Sign” [18] and the axial stress test [19]. A distal radial migration of 3 mm or greater was considered indicative of longitudinal instability [20].

After the confirmation of acute presence of Essex Lopresti syndrome, the surgery was performed with a preliminar positioning of an infraclavear catheter for continuous post operative analgesia. Patients were placed in supine position with a pneumatic tourniquet at the limb’s root.

The surgical repair was performed in three steps. Since ELI is a non frequent lesion, not all the three steps were performed in all cases, reflecting the progressive and recent development of knowledge in this pathology.

**Fig. 5:**
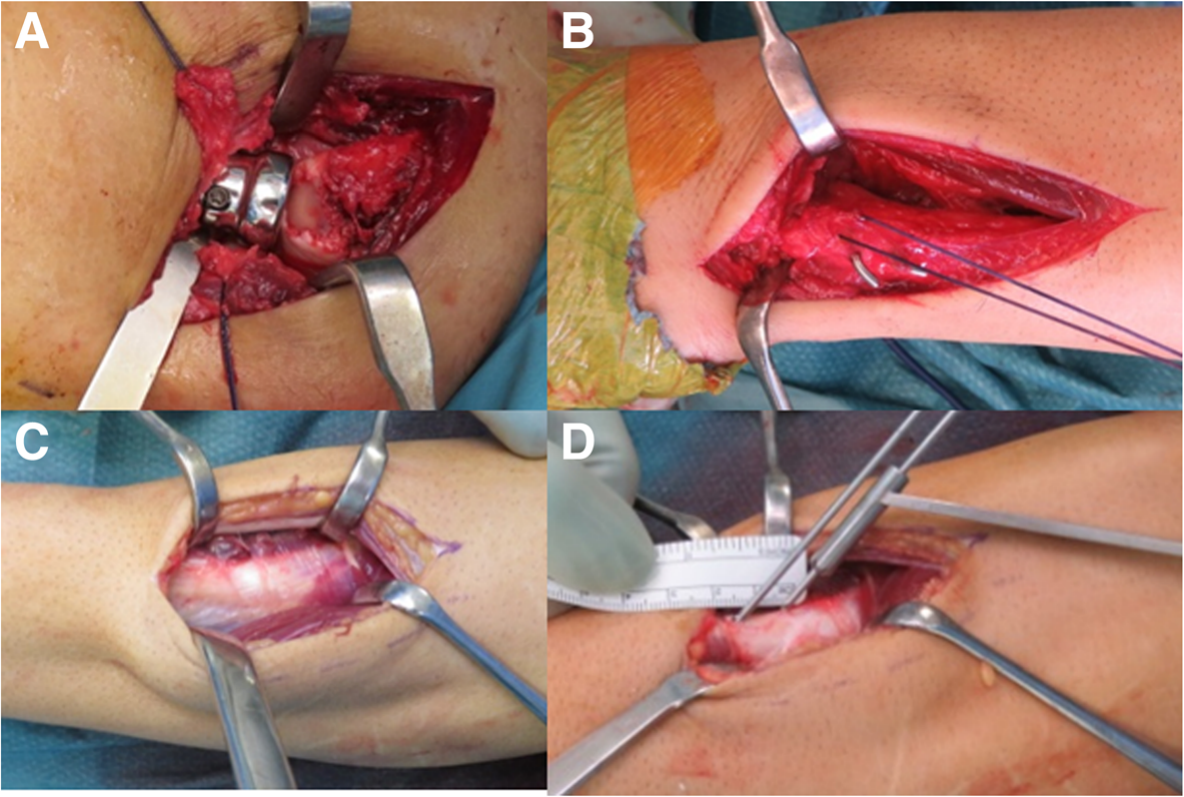
Surgical images of the procedure, clinical case 3. The radial head prosthesis was firstly positioned (**a**), followed by TFCC reconstruction and DRUJ pinning (**b**). At the level of the maximum radial bow, passing between flexor and extensor muscles, the radial origin of the pronator teres was recognized and isolated (**c**). At intermediate forearm rotation two 1.5 mmm drill were performed (**d**)

The first step, performed in all cases, consisted in the positioning of the radial head prosthesis. Using the Kocher interval the implanted prosthesis was unipolar in three cases and bipolar in two cases, all non cemented with press fit insertion in the radial canal (Fig. 5a).

In patient n.1, only radial head replacement was performed. Patient n.2 was initially underestimated: in emergency room it was classified as isolated Mason 3 radial head fracture and addressed for surgery. It was only under anesthesia and under C-arm view that forearm longitudinal instability was detected. The muscular hernia sign was negative, Axial test positive with a stable DRUJ. The radial head prosthesis was positioned, then IOM and lateral collateral ligaments reconstruction were performed. In Patients n.3 and 4 underwent radial head replacement, TFCC reconstruction, DRUJ pinning and IOM reconstruction. Patient n.5 underwent radial head prosthesis, TFCC reconstruction and DRUJ pinning.

**Fig. 6:**
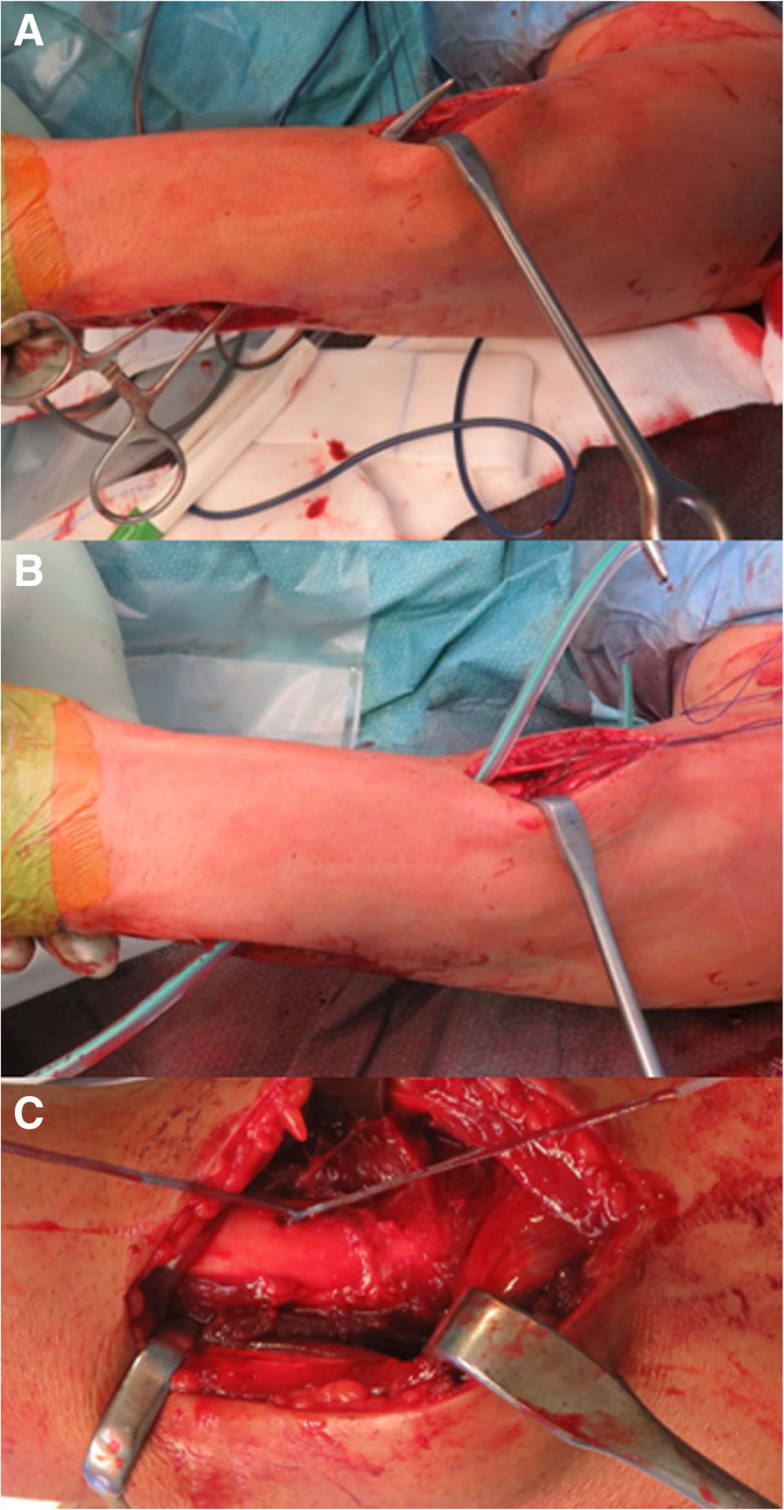
Surgical images of the procedure clinical case 3. With the help of a smooth tool the path for the stabilizer device was performed, dorsally crossing the forearm bones under the muscular extensor compartment (**a**). The stabilizer device was then put in position with the help of a knee ligament passer (**b**) and finally tensioned (**c**)

**Fig. 7:**
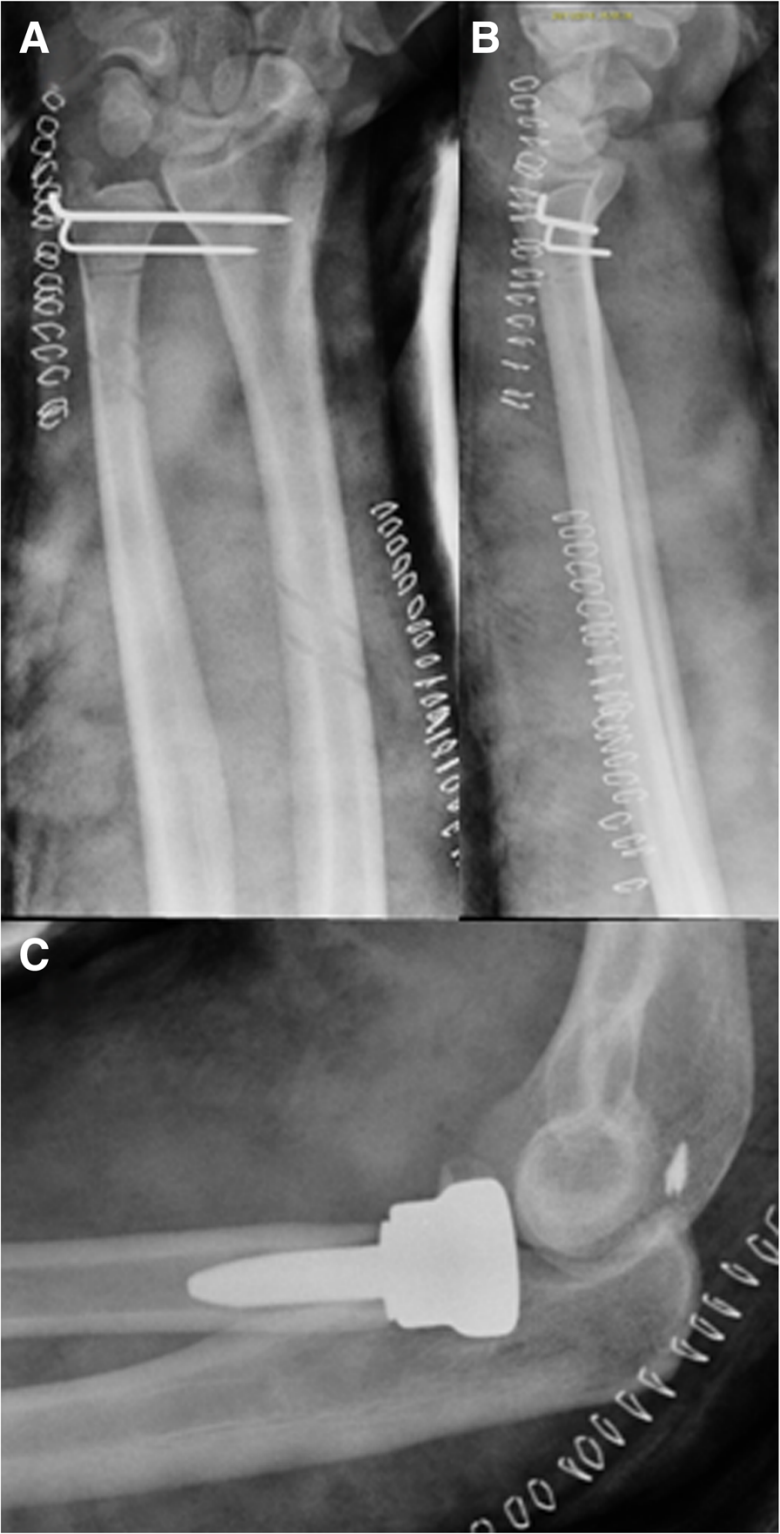
Post operative X-rays, clinical case 3. Post operative X-rays show the reduced and stabilized DRUJ (**a**, **b**) and the radial head prosthesis (**c**). It is possible to see the radial and ulnar tunnels of the two bundles of the newly reconstructed IOM (**a**)

In cases when TFCC reconstruction and DRUJ pinning were performed a dorsal access to the DRUJ was used. The TFCC was re-inserted with a high resistance 0 wire to the ulnar stiloid process with a trans osseous stitch, and the DRUJ was then reduced and fixed by two extra articular Kirschener wires. (Fig. 5b). When a IOM reconstruction was performed (patients n.2,3 and 4) it was used a technique similar to Soubeyrand procedure [21] (Figs. 5, 6, 7): at the level of the maximum radial bow and at the opposite part of inner ridge detected under C-arm, a five centimetres incision was performed. Passing between flexor and extensor muscles, the radial origin of the pronator teres was recognized. Keeping the forearm in neutral pronation and supination position, two 1.5 mm drill holes were performed. Other two 1.5 mm drill holes were performed at the level of the distal ulnar neck, with a 20 degrees axis respect to longitudinal forearm axis. As stabilizer device a cadaveric tendon allograft was used in one case (n.4) and a synthetic band (Ultratape, Smith & Nephew,UK) in two cases (patient n.2 and 3). The stabilizer device was then passed, dorsally crossing the forearm bones under the muscular extensor compartment, with the help of a plastic knee ligament passer. Under C-arm view the device was then stretched; pronation supination and radial head pistoning were checked and definitively fixed. Due to LUCL laceration observed, all patients underwent a final LUCL proximal reinsertion and in one case a MCL was proximally reinserted with metallic anchors.

All the patients underwent a post operative cast immobilization for 48 h, followed by progressive passive and active elbow and wrist mobilization. Progressive muscular reinforcement protocol was permitted starting one month after surgery. The DRUJ K wires were surgically removed after 40 days.

All patients of this study have been clinically evaluated at a mean of 17 months of follow up using the Mayo Elbow Performance Score [22] and the Modified Mayo Wrist Score [23]. An X ray investigation has been performed in all cases at final follow up.

## Results

Only few reports are present in literature about acute ELI (Table 1): Grassmann et al. [24] identified 12 acute ELI in a group of 295 patients affected by RH fracture. An evident radio-ulnar X-ray discrepancy was found in only five patients, and a partial or complete IOM rupture was diagnosed by MRI in all 12 cases. The authors reported good mid-term results. Trousdale [15] reported a case series of 20 ELI, identifying 5 cases of acute forms: these cases, properly treated, reported good outcome in 4 cases, while the other 15, initially misdiagnosed and treated with RH resection, developed severe pain at distal DRUJ, with good results even after treatment only in 3 cases. In 1987 Edwards and Jupiter [10] reported on 7 patients, 4 operated within one month, with excellent results obtained only in the three cases. The only poor result was experienced by the patient who underwent a RH excision. Duckworth [25] retrospectively reviewed 60 patients affected by RH fracture, identifying 22 patients with radio-ulnar discrepancy. The good short term results (6 months) even after conservative management are to be considered non indicative, since usually patients experience a later worsening and no indication is reported about IOM assessment. The most representative case series have been reported by Schnetzke in 2017 [14]: outcome of 16 acute and 15 late ELI were compared. Acute ELI, treated with DRUJ pinning and no IOM reconstruction) showed better clinical and radiological results, with lower rate of reoperations. The authors highlighted how seven patients had a proximalization of more than 2 mm at final follow up, associated with worst outcome: the authors conclude that this observation supports the idea that IOM is not able to heal, and once disrupted the muscle herniation through the laceration prevent its healing [24, 26].

**Table 1:** Studies in literature reporting cases of acute Essex-Lopresti injuries

Author	N. of patients operated within 4 weeks	mean follow up, months	MEPS at follow up	MMWS at follow up	mean DRUJ at final FU	Described results
Grassmann et al. [24]	12	59	86.7	88.4	not reported	
Trousdale et al. [15]	5	54	91	80	+ 2.5 mm ulna	
Edwards and Jupiter [10]	5	18	not reported	not reported	+ 2 mm ulna	3 excellent, 1 good, 1 poor
Schnetzke et al. [14]	16	63.6	91.3	81.3	+ 2 mm ulna	

Case n.1 during the post operative rehabilitation protocol complained the onset of persistent wrist pain associated to DRUJ discrepancy of 3 mm (MEPS score 72, MMWS 75), which led to ulnar shortening osteotomy 9 months after the first surgery, with good results at final follow up (MEPS 83, MMWS 88) (as reported in Table 3).

The complete data set is reported in Tables 2 and 3.

**Table 2:** Patients’ demographics and lesion characteristics

Patient n.	Name	age	sex	Injury type	Mason grade	DRUJ discrepancy mm	M hernia sign	Axial test	Essex-Lopresti Injury Type
1	PA	41	M	road traffic accident	3	3	+	+	Acute engaged
2	RM	46	M	bike fall	3	0	–	+	Undetected at Imminent Evolution
3	FDR	33	M	fall while walking	3	7	+	+	Acute engaged
4	CM	42	M	road traffic accident	4	9	+	+	Acute engaged
5	GS	40	M	bike fall	4	6	+	+	Acute engaged

**Table 3:** Patients intra operative and clinical data set

Patient n.	RH prosthesis	IOM plasty	IOM plasty material	TFCC reinsertion	Reoperation	FInal Follow Up time	MEPS at follow up	MMWS	DRUJ at final FU
1	yes	no		no	Ulnar shortening at 9 months	16	83	88	+ 2 mm ulna
2	yes	yes	Ultratape	no	no	15	95	95	+ 0 mm
3	yes	yes	Ultratape	yes	no	15	92	94	+3 mm ulna
4	yes	yes	Allograft	yes	no	25	100	85	+ 2 mm ulna
5	yes	no		yes	no	14	90	92	+ 0 mm

## Discussion

In 2007 Marc Soubeyrand proposed the “Three Forearm Constraints” concept [27]. The Forearm Unit has to be considered like an association of three main constraints: the PRUJ, the IOM and the DRUJ. Each constraint is essential for stability and movements of the forearm. In case of single constraint damage (distal radius fracture, simple RH fracture, and so on) a pronation-supination decrease occurs, without causing instability (Stage 1). In case of two constraints damage (Stage 2) a partial transversal instability may occur (Criss-Cross lesion, Galeazzi lesion, Monteggia lesions). The disruption of three constraints (Stage 3) causes a longitudinal-transversal instability (Acute). Stage 2 and 3 patterns present an intrinsic instability, and may be grouped under the “Unstable Fractures of the Forearm” definition. In this different conditions, the lack of at least two of the three constraints (TFCC, IOM and RH) can lead to two different patterns. The first is an acute and evident longitudinal instability of the forearm, defined by the authors “Acute Engaged Essex-Lopresti Injury”. The second has already been identified by different Authors [7, 9, 10, 15] but still not pointed as specific clinical entity: it shows a more obscure clinical pattern, easy to misdiagnose but still causing instability, defined by Authors “Undetected at Imminent Evolution Acute Essex-Lopresti Injury”, observed also in the two patients reported by Helmerhorst et al. [28]. Usually a correct diagnose is performed in chronic setting, when the symptoms of a longitudinal instability became evident but unfortunately with poor outcome [14]. The clinic extrinsication of one of these two conditions depends on the IOM answer to the trauma. In the first case, an immediate proximal translation greater than 5 mm associated with impacted RH fracture into Capitulum Humeri is significative for acute high-henergy complete irreparable IOM and TFCC laceration. Aim of this work was to examine the different lesion patterns that may cause forearm instability, focusing on cases treated by the authors and the few literature reports, in order to better define the different entities.

Among the cases enrolled for this paper, the Authors observed four cases presenting characteristics of Acute Engaged ELI. Unfortunately not all patients received the same treatment: due to the rarity of this condition the knowledge development on anatomopathology and treatment is still ongoing, so it is only in the recent years that it has been properly understood, diagnosed and treated. It is very important to pinpoint how most of the authors indicate that this lesion seems to occur more often than realized up to now, reporting values of around 38% of correct diagnoses performed in excellence centers [8]. Similarly to other series reported in literature, the cases treated with RH implant, IOM reconstruction and TFCC fixation and pinning reported higher scores and better functional outcomes, whereas the patient who underwent the isolated radial head replacement reported worst outcomes, requiring a shortening ulnar osteotomy to treat the persistent wrist pain. It was noticeable that in this case after the RH replacement a proximal radial migration was barely evident with a DRUJ discrepancy of 3 mm, and the treatment seemed to be sufficient with the experience maturated at that time. A possible explanation is due to a partial IOM/TFCC tear caused by the high-energy trauma, that became complete after repetitive tractions by Biceps Brachii. This condition progressively evolves into a proximal radial migration causing DRUJ instability-discomfort and grip weakness.

**Fig. 8:**
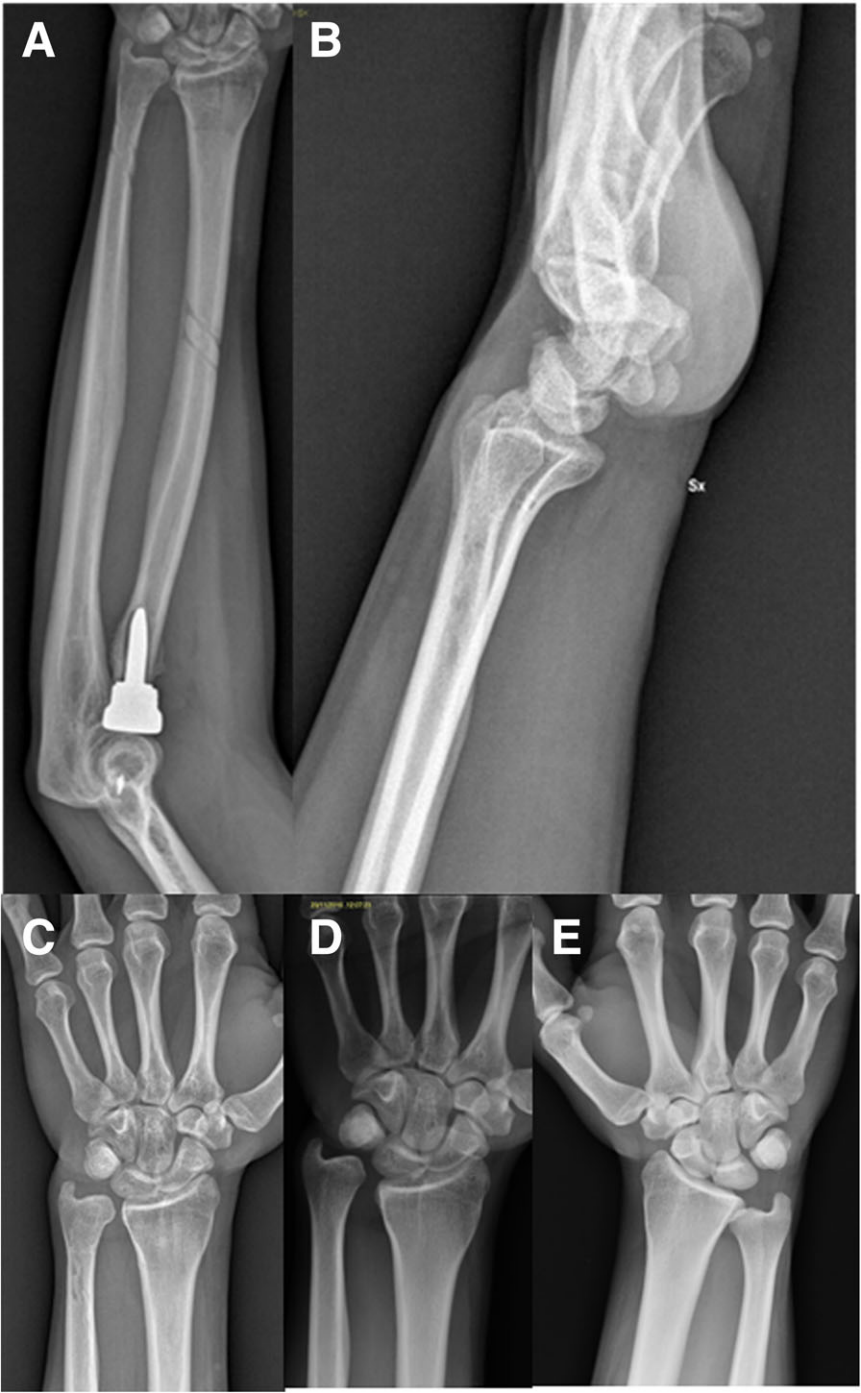
1 year X-rays, clinical case 3. Follow up X-rays at 1 year of follow up, showing the radial head prosthesis in situ and the whole forearm (**a**). The lateral view shows no dorsal dislocation of the distal ulna (**b**). At the DRUJ a slight recurrence of the ulnar plus is evident (**c**), even if non symptomatic. Nevertheless the improvement pre-operative wrist x-ray (**d**) is evident. **e** Image shows the opposite side normal wrist

**Fig. 9:**
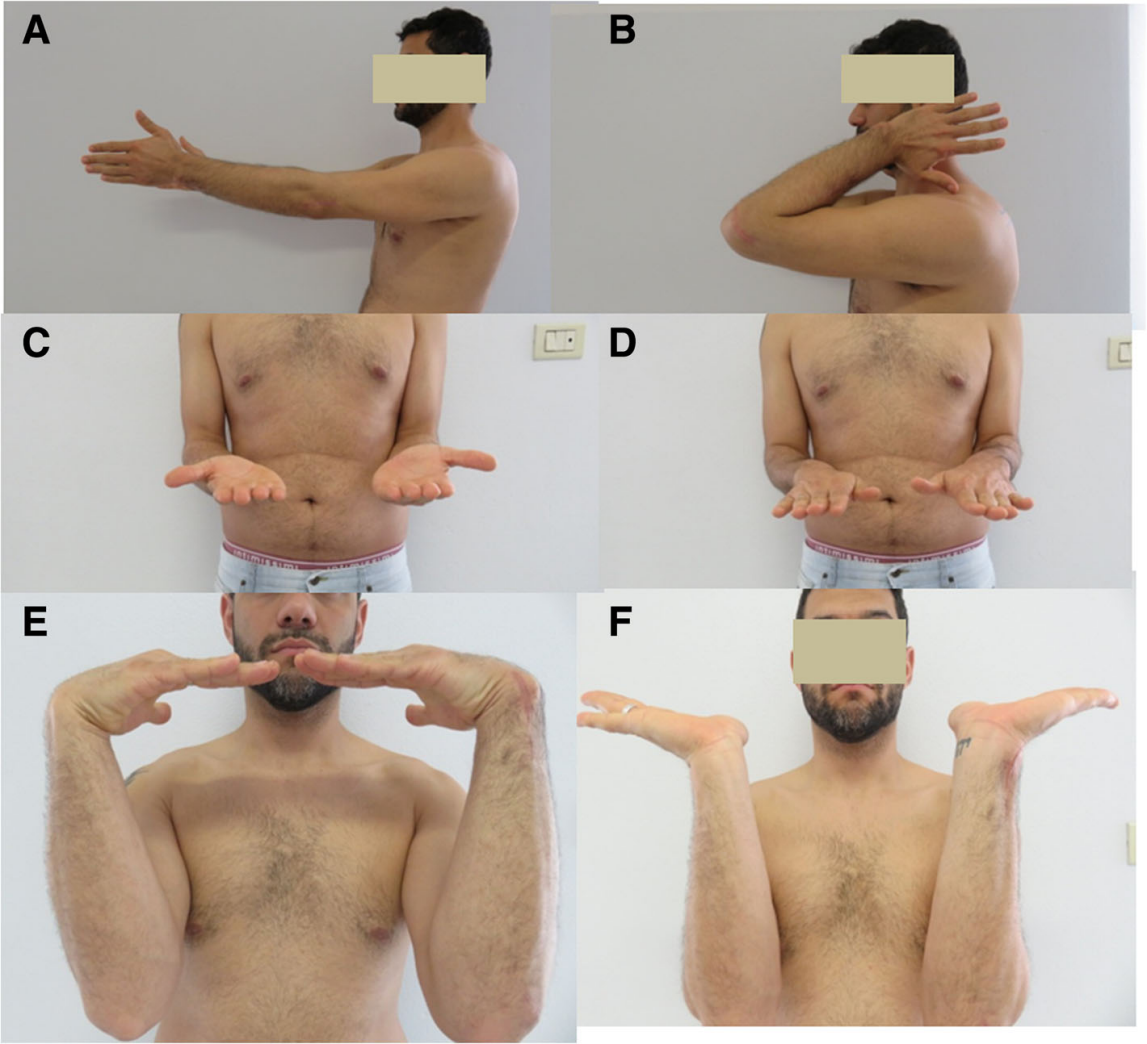
Clinical follow up, clinical case 3. Follow up clinical aspect at 1 year of follow up, showing a good movement of the elbow (**a-d**) and the wrist (**e**, **f**)

These observations lead to the confirmation that there is an elevated possibility to misdiagnose these non evident acute Essex-Lopresti, that in a first step may be considered and treated as simple RH fracture but shortly express the typical symptoms of a forearm instability. Basing on several observation of similar cases, in 2006 in fact Junghbluth et al. introduced the term “missed” Essex-Lopresti [12], characterized by a painful but correctly positioned radial head prosthesis in a context of longitudinal instability of the forearm due to IOM laceration. Patient n.3 (Figs. 2, 5, 6, 7, 8, 9) experienced a progressive worsening of DRUJ discrepancy at follow up compared to post operative control: this may be explained with a slight tension loosening of the IOM and DRUJ reconstruction. At the final follow up this condition was non-symptomatic, supporting the idea that if left untreated the clinical results were prone to deteriorate even more at follow up, as observed in case n.1. These results are consistent with the few reports available in literature (Table 1), with comparable values of MEPS, MMWS and DRUJ discrepancy at follow up. The higher clinical results have been obtained in cases when the IOM have been reconstructed, highlighting the importance of this anatomical structure.

For these reasons it is mandatory to perform an accurate clinical examination to the patient in acute setting, tagging these cases as Undetected at Imminent Evolution ELI and addressing them to a proper and complete treatment. The diagnosis of the acute engaged pattern of ELI is easier to recognize. On the other side, a radial translation inferior to 5 mm associated with Mason 3 radial head fracture, forearm or wrist painful and positive radiological Axial Test is to be considered indicative for an acute IOM laceration, even if not as evident as the acute engaged pattern presentation. Therefore, the diagnosis of Acute Undetected at imminent evolution ELI is difficult, because a proximal radial translation inferior to 3 mm does not lead to an immediate longitudinal instability [29]. Imaging does not give an effective contribution, so the clinical investigation part and the physical examination are fundamental for the correct diagnosis. The main limitation of this study is represented by the low number of cases, mainly because ELI is an uncommon condition. This led to a consequent limitation, that is the different surgical procedure performed and the different approach to ELI. At the same time this reflects the development in the knowledge of this disease over the last years.

## Conclusions

From the analysis of literature and the presented case series, ELI can be considered part of the unstable fractures of the forearm, where a radial head fracture is associated to one or more ligament lesions. This is an acute lesion in all cases, that may be evident (Engaged) or at imminent evolution (Undetected) due of the complete or partial rupture of at least two of the three constraints of the forearm unit. The scarce frequency and the poor diagnostic tools make the early diagnosis of the Undetected form very challenging. The comprehension of the lesion dynamics, the accurate anamnesis and clinical investigation with proper tests and the help of a well-conducted X-ray and CT examination may lead to these lesions early detection and treatment with a better outcome. The surgical technique has to follow some progressive steps: the radial head has to be replaced first, then the stability has to be re tested and in case of instability the DRUJ has to be reconstructed along with fixation of the TFCC. The final step should be the IOM reconstruction to give the proper tension to the construct.

## Abbreviations

DOB: Distal Oblique Band
DRUJ: Distal Radio-Ulnar joint
ELI: Essex-Lopresti Injury
IOL: Interosseous ligament
IOM: Interosseous Membrane
PRUJ: Proximal Radio-Ulnar Joint
RH: Radial Head
TFCC: Triangular Fibrocartilage Complex
